# A Counseling Application as an Alternative Tool in Increasing Coping Self-Efficacy Among University Students With Academic Distress During Coronavirus Disease 2019 Pandemic in Indonesia: A Study Protocol for a Randomized Controlled Non-Inferiority Trial

**DOI:** 10.3389/fpsyg.2021.712806

**Published:** 2021-10-28

**Authors:** Zahrotur Rusyda Hinduan, Miryam Wedyaswari, Ilham Phalosa Reswara, Hari Setyowibowo

**Affiliations:** Center for Psychological Innovation and Research, Faculty of Psychology, Universitas Padjadjaran, Bandung, Indonesia

**Keywords:** coping self-efficacy, resilience (personality), depression, mobile-based counseling application, technology-based intervention, non-inferiority controlled trial, solution-focused brief therapy

## Abstract

The coronavirus disease 2019 (COVID-19) pandemic increased education-related distress among University students globally, including in Indonesia. Psychological factors, such as academic demands, limited opportunity to meet their peers, problematic use of technology, and domestic problems, influenced the well-being of the students, leading to poor academic performance. A mobile-based counseling application was developed to address the distress among University students. The application was meant to reach students living remotely to enable them to access psychological assistance. Therefore, the purpose of this study was to describe a protocol aimed to evaluate the equivalence of the application when compared to the Treatment-As-Usual (TAU) in increasing the coping self-efficacy (CSE) and resilience of students as well as in decreasing their level of depression. A two-armed parallel randomized control non-inferiority trial will be conducted among approximately 430 students with selected academic problems. The participants will be randomly allocated into the TAU and the intervention groups. The primary and secondary outcomes will be measured by the Indonesian versions of the Coping Self-Efficacy (CSE) Scale, the Resilience Scale (RS-14), and the Patient Health Questionnaire (PHQ-9). The data will be collected at baseline, at the end of each session, and after 3 months. The outcomes will be analyzed using repeated-measures ANOVAs, intention-to-treat, and per-protocol analysis. If proven, the application will be used as an alternative media in helping the students.

**Clinical Trial Registration:** Thailand Clinical Trials Registry (TCTR20200530001); Date of registration: May 28, 2020.

## Introduction

The coronavirus disease 2019 (COVID-19) pandemic influenced many aspects of human health as follows: infecting many people, causing severe disease, and increasing mortality rate, especially among vulnerable people (World Health Organization, 2020). Also, the pandemic disrupts other aspects of human life, since the virus is transmitted between people through indirect, direct, and close contact. Therefore, the human movement was limited, such as social and economic activities. People stayed at home to avoid contact with other people, though this social isolation may lead to adverse psychological conditions. According to Tull et al. (2020), people tend to be anxious, worried, and lonely under stay-at-home order. Another study showed that during this situation, there are significant changes in the sleep and work patterns of people. The total sleep time and screen time decrease and increase, respectively, thereby affecting their moods significantly (Conroy et al., 2021). Park et al. (2020) stated that the pandemic increases the uncertainty that affects well-being and stress.

Furthermore, several studies conducted during the pandemic showed that the prevalence of mental health issues among University students has increased (Chen et al., 2020; Son et al., 2020). The majority of respondents perceive stress, anxiety, and depression. There are several sources of stress (Misra and Castillo, 2004; Stevenson and Harper, 2006), such as (1) worrying about health conditions; (2) feeling isolated; and (3) concern about the academic workloads and performances. The academic-and non-academic-related stress causes several problems among the students though not limited to lowered grades and dropped courses (The American College Health Association, 2009). Also, it reduces well-being (Misra and Castillo, 2004; Stevenson and Harper, 2006).

Personal resources, such as self-mastery or coping self-efficacy (CSE), are important in lowering distress and apprehension during the pandemic (Ben-Ari and Ben-Yaakov, 2020). Generally, CSE refers to the confidence in one's own ability to carry out various coping strategies and execute a course of action designed to manage an external stressor (Watson and Watson, 2016). This personal resource aligns with the self-efficacy concept (Bandura, 2002), which leads to having better effort, persistence, and resistance. Moreover, the CSE enables students to exhibit positive strategies to manage adversities in their lives, including during the pandemic (Watson and Watson, 2016). A study showed that students with better CSE have better adjustment and less stress (Denovan and Macaskill, 2013).

Generally, the studies of students suggested the need for the support of universities to help manage stress during the long-lasting pandemic being studied (Pajarianto et al., 2020; Son et al., 2020). The interventions need to fit the stay-at-home situation and the characteristics of young people. Consequently, the University students are categorized as Generation Z, who were born between 1995 and 2000 (Hampton and Keys, 2017). They become familiar with the digital world at a young age and, hence, are the most electronically connected generation (Geck, 2006). The students are already getting used to communicating, collaborating, or interacting through the Internet (Hampton and Keys, 2017). Also, these characteristics are true for Generation Z in Indonesia (Hinduan et al., 2020). Among the electronic devices, the smartphone is the most frequent device used every day, and it accounted for 3.5 h/day before the pandemic (Zaenudin, 2017). The Internet, especially smartphones, is a promising media to focus on in designing the new intervention effective for University students during the pandemic.

According to several studies, online counseling is needed by students because they cannot access mental healthcare (Chan, 2020; Li and Leung, 2020; Novella et al., 2020). Although there are several limitations, the studies showed that online counseling is effective in dealing with psychological issues such as emotional distress, anxiety, and social withdrawal symptoms. The study aimed to test whether or not the mobile-based counseling application called “SAENA” can help the students increase their (1) CSE and (2) resiliency, as well as decrease their (3) level of depression. Therefore, the hypothesis is that the application is equivalent to the Treatment-As-Usual (TAU) in increasing the personal resources of students.

The quality of a study depends on how well-both the design and execution phases of the project are (Ott, 1991). By writing the protocol, a gap between these phases could be examined carefully and fairly. The protocol also helps the researchers in providing study documentation as well as in increasing the efficiency of the projects (Ott, 1991). Therefore, this study will explain the protocol of the non-inferiority trial of the mobile-based counseling application.

## Materials and Methods

### Study Design

This study describes a study that will be conducted as a two-armed parallel randomized controlled non-inferiority trial. The trial is in line with the guidelines for the protocol content of the non-inferiority trial (Piaggio et al., 2012). It will also be based on the guidance of the Standard Protocol Items: Recommendations for Intervention Trials (SPIRIT) Statement (Tetzlaff et al., 2012; Chan et al., 2013). The non-inferiority scheme will be used in this study because the application is not aimed to be more powerful or effective than the usual treatment but aimed to increase the practicality of the counselors to store the data in the application and to hide personal contacts (e.g., email addresses or online meeting IDs) by solely using the application only for the counseling purposes (Hahn, 2012).

To eliminate the assay sensitivity, bias, and constancy assumption (ABC) of this non-inferiority trial, there are several things included in the design. First, the outcome measurement tools that will be used in both groups are reliable. Second, besides using randomizing in allocation and double-blinding procedure, the control group will get the TAU without any intervention to prevent a bias. Third, the data will be collected in the same period for both groups to minimize any situational effects, such as the COVID-19 pandemic-related government policies in this country (Walker, 2019).

**Figure 1:**
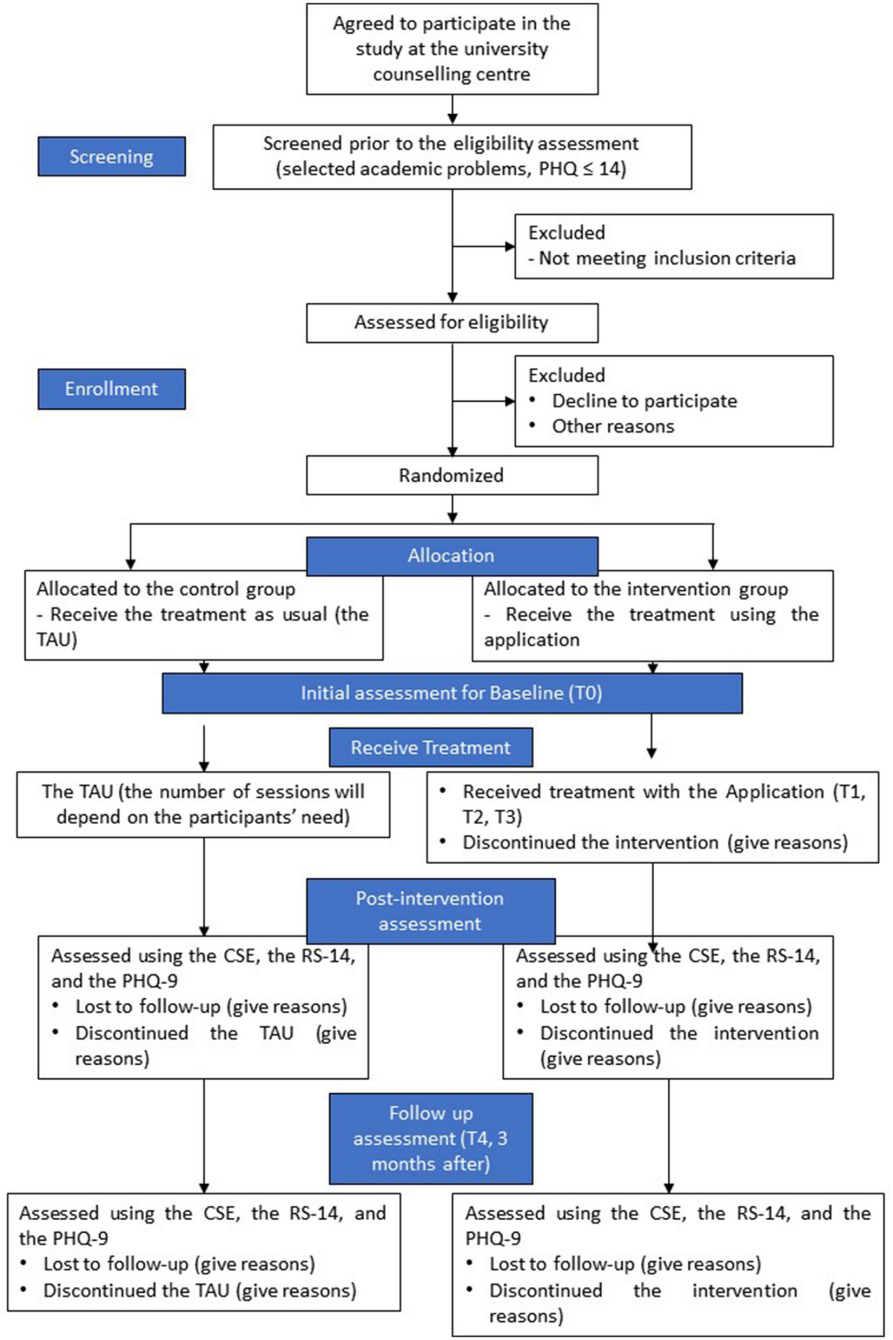
Study design.

The proposed study will be a parallel group design comparing two types of treatments. The intervention group will use the application whereas the control group will get the TAU. The intervention group will have three counseling sessions for a month; meanwhile, the control group will have many sessions based on the need of each individual. All outcomes will be assessed repeatedly using online self-reported forms, which will be facilitated by research assistants. The assessments will be taken (1) before the intervention, (2) after each session (a session/week), and (3) after 3 months. The study design is shown in Figure 1.

This study has been approved by the Research Ethics Committee of the Universitas Padjadjaran, Indonesia (No.1455/UN6.KEP/EC/2019) and has been registered in Thailand Clinical Trials Registry (TCTR; Identifier: TCTR20200530001). Protocol amendments will be updated in the trial registry (TCTR) so the study can be examined by any other parties.

### The Mobile-Based Counseling Application: SAENA

The application was developed in four major steps as follows: First, the application and a counseling protocol were developed. The application facilitates the helping relationship between the students and the counselors through written texts, voice calls, and video calls. These three communication modes can be used interchangeably during counseling sessions. The counseling protocol was developed based on the solution-focused brief therapy (SFBT) approach (Bavelas et al., 2013). The approach is effective in a lot of contexts (Kim, 2008; González Suitt et al., 2016), except for complex psychological problems (Chaudhry and Li, 2011). As a result, in the study, the participants will be screened using the Patient Health Questionnaire (PHQ-9) to exclude those with severe depressions. Second, the professionals who will perform the counseling were trained in using the application. However, the counselors need to have a prior experience in conducting micro-skills in a face-to-face counseling context. Third, a feasibility study was conducted. Based on this study, several improvements were made in the application and the protocol. Finally, the protocol of the non-inferiority trial was developed.

### Participants

The target population in the study will be University students who have academic-related psychological problems. The inclusion criteria of the sample are a University student, aged 18–25 years old, having academic-related problem(s), and registering as a participant in this study. The academic-related problems that will be included in the study are (1) (educational) adjustment difficulties; (2) difficulties in writing a thesis; (3) academic procrastination; (4) poor self-regulation for learning; and (5) a low grade point average (GPA). The study will exclude students with (1) physical disabilities; (2) under the care of mental health professionals; and (3) the score of PHQ-9 more than 14 (having severe depression), (Rahmadiana et al., 2019).

The participants of the study will be those who register in a University counseling center. They will be then screened with the PHQ-9. If they are willing to participate in the study, the research assistants will give informed consent and the right to withdraw from the study at any given time they wish to. The students who decline to join the study or withdraw from the study will be followed up to identify their reasons for declining and withdrawing from the study. The students have a right to undergo post-trial treatment if needed.

The sample size is a sensitive issue in the non-inferiority trial (Hahn, 2012). Large sample size is usually needed. However, if the new intervention is assumed to be slightly more effective than the TAU, the number of samples can be reduced. The mobile-based counseling application can be assumed to be more practical for the students in this country. Those who live remotely can still access the psychological services. The data from the University counseling center in 2020 were also a source of consideration in determining the sample size. The center admitted 482 clients in that year, with an average of 40 clients per month. With 80% CI, the expected SD will be one, and the inferiority margin (Δ) will be 0.24. It was calculated that the number of participants per arm needs to be 215 and the total sample size needs to be 430.

### Randomization

The participants who will be randomized are the students who register as clients at the University counseling center, meet the inclusion criteria, and are willing to participate in this study. The randomization will be carried out by the research assistants using a computerized random number generator. The results from the randomization are a list of registration numbers that will be categorized as the intervention group and the control group. The participants will be then assigned to those two groups based on their registration numbers. The allocation will be performed before the initial assessment.

### Outcome Measurements

The participants in the intervention group will complete the assessments at (1) the baseline (T0); (2) at the end of the first counseling session (T1); (3) at the end of the second counseling session (T2); (4) at the end of the third counseling session (T3); and (5) 3 months after the third counseling session (T4). There will be a week interval between the counseling sessions (T1, T2, and T3). Meanwhile, the participants in the control group will be measured at 3–5 points in time depends on the number of counseling sessions they have. They will fill the questionnaires at (1) the baseline (T0); (2) at the end of their counseling session(s), (T1 and/or T2 and/or T3, etc.); and (3) 3 months after the last session (TX). The data will be collected using the online forms and will be assisted by the research assistants.

The primary outcome of the proposed study will be CSE measured by the Coping Self-Efficacy (CSE) Scale. The Indonesian version of the CSE consists of 26 items with 11-point scale (0–10). The scale has good reliability (*r* = 0.92), (Nurhidayah, 2020). The total score will be used as an indicator of the level of CSE of participants. The CSE is defined as a belief about own ability of an individual to perform specific coping behavior (Chesney et al., 2006). Self-efficacy serves as evidence in measuring changes in the coping ability of individuals, especially for those who seek professionals to solve their problems (Chesney et al., 2006; Yoo et al., 2020).

The secondary outcomes of this study will be (1) resilience and (2) level of depression. Resilience is defined as the capacity of a dynamic system to adapt successfully to adversity that threatens system function (Masten, 2014). The Resilience Scale (RS-14), (Surzykiewicz et al., 2019), which will be used to measure this variable, has five dimensions, namely, equanimity, perseverance, self-reliance, meaningfulness, and existential aloneness. The short version of the RS test has been adapted to the Indonesian language. It has 14 items with a 7-point Likert scale (*r* = 0.87). The scale has good Construct Validity Fitness Indexes [Non Normed Fit Index (NNFI) = 0.99, Root Mean Square Error of Approximation (RMSEA) = 0.027, and Standardized Root Mean Residual (SRMR) = 0.026], (Valentino, 2021).

The PHQ will be used to measure the level of depression. The Indonesian version of this scale has nine items with a 4-point Likert scale (*r* = 0.80), (Onie et al., 2020). Similar to other scales, the PHQ-9 is a self-report questionnaire. The total score will be used as a cutoff score in the initial screening. A total score of 15 or higher indicates moderate and severe major depression that can be an indicator for initiating a drug treatment (Arroll et al., 2010). The students with a PHQ-9 score of 14 or lower will be included in this study. The results from this scale will also serve as the secondary outcome of this study.

The demographic data of participants will also be collected using the form. Some of the data will be used by the counselors for intervention purposes (e.g., family, educational, and organizational background) while the rest will be used only for research purposes (e.g., gender, ethnicity, age, University level, and university major).

The main researchers will develop a data management form, and the research assistants will input all the data. The identities of participants will be coded to maintain confidentiality. The data management form also has a password, which is known only by the researchers.

### Determining an Inferiority Margin of the Trial

In the non-inferiority trial, the researchers need to determine Δ (Hahn, 2012). In this study, the margin is the maximum acceptable extent of non-inferiority of the mobile-based counseling application. The maximum limit of Δ can be set to the entire effect size of the active control treatment (Hahn, 2012). Several proposals for choosing the inferiority margins in the context of SFBT have been made (Table 1). There are several suggestions for the maximum difference in outcomes that will be considered clinically irrelevant. It ranges from *d* = 0.17 to *d* = 1.76. The smallest margin was suggested by Schmit et al. (2016), (*d* = 0.24) for the treatment of internalizing disorders. Thus, in this study, it was decided to use the inferior margin of 0.22 (i.e., the inferior margin up to 0.22), corresponding to a small effect size.

**Table 1:** The effect size from the solution-focused brief therapy (SFBT)-related studies.

**Previous studies**	**Effect size**
Impacts on academic and emotional difficulties (Daki and Savage, 2010)	≈0.13 (partial eta squared, medium effect size)
Meta-analysis of solution-focused brief therapy for treating symptoms of internalizing disorders (Schmit et al., 2016)	≈0.24 (Hedges' g, small effect size)
Examining the effectiveness of solution-focused brief therapy: a meta-analysis (Kim, 2008)	0.26–0.38 (Cohen's *d*, small effect size)
SFBT for health-related psychosocial outcomes (Zhang et al., 2018)	0.34 (Cohen's *d*, small effect size)
Solution-focused brief therapy in schools: A review of the outcome literature (Kim and Franklin, 2009)	0.38–1.76 (Cohen's *d*, medium to large effect size)

### Data Analysis

Descriptive statistics will be used to describe relevant demographic data, such as the frequency and the percentage for the categorical data as well as the mean and the SD for the outcome measurements. There will be three analysis plans to prove the non-inferiority of the application, which are (1) repeated-measures ANOVAs (Goodman et al., 2016; Twisk et al., 2018), which will be used to test for differences between the TAU and the intervention groups (i.e., between-subjects factor) in the treatment at the four time points (i.e., within-subjects factor). We would like to assess whether the outcomes will be improved after treatment using the application and the TAU. An analysis will be conducted to check the constancy assumption. Then, both (2) the intention-to-treat (ITT) and (3) the per-protocol analysis will be performed. Previous studies showed that performing both analyses will produce more convincing results to conclude the non-inferiority of a new treatment (Hahn, 2012; Walker, 2019). The ITT analyses will be chosen to handle the missing value due to the protocol deviations, withdrawal, and non-compliance (Gupta, 2011) whereas the per-protocol analysis will minimize the factors that would make the two groups seem similar (Walker, 2019).

The statistical hypotheses in this non-inferiority trial use Δ. The null hypothesis states that the intervention using the application will be inferior to the TAU (H0: TAU–SAENA ≥ Δ). The alternative hypothesis states that the intervention will not be inferior (will have a similar effect) to the TAU although the effect may not be as good as the TAU within the extent of margin (H1: TAU–SAENA < Δ). Of note, 95% CI will be used. The interval will be the mean difference between the outcome measures of the two groups. The formula is as follows:


$$\begin{array}{l} \left(\mu_{saena}-\;\mu_{TAU}\right)\pm 1.96\sqrt{\frac{\sigma_{saena}^{2}}{n_{saena}}+\frac{\sigma_{TAU}^{2}}{n_{TAU}}} \end{array}$$

μ, mean; Tau, standard deviation; *n*, sample.

The 1.96 is the *z*-value, and the value from this formula can now be compared with Δ. If the interval remains above Δ, then the inferiority of the application will be proven according to the standards that have been set. In contrast, if the upper limit for treatment difference by the intervention group compared to the TAU group is < Δ, then the non-inferiority will be concluded.

## Discussion

This study will provide the initial evidence of the equivalence of the mobile-based counseling application in improving the CSE and resilience as well as decreasing the level of depression among students with academic problems during the pandemic. While academic distress during the pandemic is the main focus of this study, the application is designed to be relevant to a broad range of mild psychological issues among young people.

The protocol modifications will be communicated after this study is performed. A trial registry amendment will be performed at the TCTR website. Since there was a limited study that tests the equivalency of a protocol in psychological intervention in Indonesia, this study would provide a standardized protocol for improving psychological aspects (i.e., the primary and secondary outcomes) among the students. Furthermore, this study would be very useful in helping people in this pandemic because it provides a protocol that requires no face-to-face condition to deliver counseling sessions.

There were several previous non-inferiority studies in the fields of online psychological intervention. The most common one is cognitive behavior therapy (CBT)-based interventions. Several studies show that the online CBT-based interventions are equivalent to the face-to-face CBT-based intervention with moderate to large effect size in helping participants to reduce insomnia and depression symptoms (Andersson et al., 2013; Wagner et al., 2014; Blom et al., 2015). Norwood et al. (2018) conducted a meta-analysis study regarding the non-inferiority trials on videoconferencing psychotherapy. The study shows that the videoconferencing psychotherapy is partly non-inferior while compared to the face-to-face psychotherapy. Moreover, a study was conducted by Mak et al. (2018) on the efficacy of a mobile application-based program on mental health. The application consisted of (1) mindfulness-based training; (2) self-compassion training; and (3) cognitive-behavioral psychoeducation. This study shows that the application-based program is efficacious in improving well-being. However, this study does not take the non-inferiority margin as a criterion to analyze the non-inferiority of treatment. As a result, this proposed study will enrich the non-inferiority evidence of the online-based SFBT.

As mentioned earlier that this kind of trial needs to have a large number of participants to ensure that the statistical power is sufficient to differentiate the two groups (Hahn, 2012). This study might be able to have the ideal number of participants due to limited resources. However, since the application-based counseling is assumed to be more practical for the young generation, based on the previous studies, we aimed for a small effect size, so the number of participants in this study might be limited but not underpowered.

## Ethics Statement

The studies including human participants were reviewed and approved by the Research Ethics Committee of Universitas Padjadjaran, Indonesia (No.1455/UN6.KEP/EC/2019) and has been registered in Thailand Clinical Trials Registry (Identifier: 94 TCTR20200530001). Protocol amendments will be updated in the trial registry (TCTR). Participants will provide their written informed consent to participate in the study.

## Author Contributions

ZH, HS, and MW arranged the concepts and design. ZH, MW, and IR performed the literature search. IR performed the data acquisition. IR and MW prepared the manuscript. ZH edited the manuscript. HS reviewed the manuscript. All authors contributed to the study conception, design, data analysis, and writing the manuscript. All authors read and approved the final manuscript.

## Funding

This study was supported by the Ministry of Research and Technology, Higher Education Republic of Indonesia (Grant No. 1827/UN6.3.1/LT/2020). The funder had no role in designing the study, collecting and analyzing the data, publishing decision, or preparing the manuscript.

## Conflict of Interest

The authors declare that the research was conducted in the absence of any commercial or financial relationships that could be construed as a potential conflict of interest.

## Publisher's Note

All claims expressed in this article are solely those of the authors and do not necessarily represent those of their affiliated organizations, or those of the publisher, the editors and the reviewers. Any product that may be evaluated in this article, or claim that may be made by its manufacturer, is not guaranteed or endorsed by the publisher.

## Abbreviations

RCT: randomized control trial
CSE: Coping Self-Efficacy
RS: Resilience Scale
TCTR: Thailand Clinical Trials Registry
SPIRIT: Standard Protocol Items: Recommendations for Intervention Trials
TAU: Treatment-As-Usual
PHQ: Patient Health Questionnaire
SFBT: solution-focused brief therapy
CBT: cognitive behavioral therapy
REBT: rational emotive behavior therapy.
